# Effect of the Thermal Treatment of Fe/N/C Catalysts for the Oxygen Reduction Reaction Synthesized by Pyrolysis of Covalent Organic Frameworks

**DOI:** 10.1021/acs.iecr.1c02841

**Published:** 2021-11-03

**Authors:** Álvaro García, Tarrick Haynes, María Retuerto, Pilar Ferrer, Laura Pascual, Miguel A. Peña, Mohamed Abdel Salam, Mohamed Mokhtar, Diego Gianolio, Sergio Rojas

**Affiliations:** † Grupo de Energía y Química Sostenibles, Instituto de Catálisis y Petroleoquímica, CSIC, Marie Curie 2, 28049 Madrid, Spain; ‡ Diamond Light Source, Harwell Science and Innovation Campus, Didcot OX11 0DE, United Kingdom; § Chemistry Department, Faculty of Science, King Abdulaziz University, P.O. Box 80200, Jeddah 21589, Saudi Arabia; ∥ Instituto de Catálisis y Petroleoquímica, CSIC, Marie Curie 2, 28049 Madrid, Spain

## Abstract

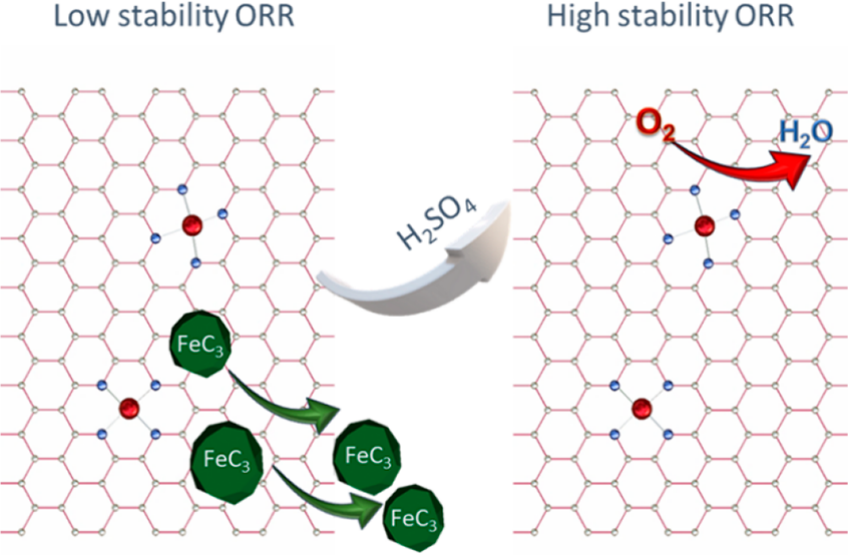

A nitrogen-containing covalent organic framework obtained from the polymerization of 1,3-dicyanobenzene has been used as a starting material for the synthesis of Fe/N/C catalysts for the oxygen reduction reaction (ORR). In this work we report the effect of the thermal treatments on the nature and catalytic properties of the catalysts obtained after the thermal treatments. After the first thermal treatment, the catalysts obtained contain metallic iron and iron carbide particles, along with a minority fraction of inorganic FeN_*x*_ sites. After acid leaching and a second thermal treatment, FeN_*x*_ sites remain in the catalysts, along with a minor fraction of graphite-wrapped Fe_3_C particles. Both catalysts display high activity for the ORR, with the catalyst subjected to acid leaching and a second thermal treatment, 2HT-1,3DCB, displaying higher ORR activity and a lower production of H_2_O_2_. This observation suggests that iron particles, such as Fe_3_C, display ORR activity but mainly toward the two-electron pathway. On the contrary, FeN_*x*_ ensembles promote the ORR via the four-electron pathway, that is, via H_2_O formation.

## 1 Introduction

Fuel cells generate electrical work by combining two redox reactions, namely, the hydrogen oxidation reaction (HOR) and the oxygen reduction reaction (ORR), therefore generating a direct electrical potential difference (work) with H_2_O as the only byproduct. Both reactions take place in the presence of Pt-based catalysts, but because of the sluggish kinetics of the ORR reaction, the loading of Pt used in the cathode is greater than that in the anode. Whereas in acid, electrolyte Pt-based catalysts are state-of-the-art ORR catalysts, in alkali, electrolyte catalysts based on transition metals can display ORR performances comparable to that of Pt/C.^1−3^ In particular, the so-called M/N/C catalysts, which are nonprecious metal catalysts (NPMCs) based on transition metals (M = Fe, Co, or Mn) coordinated to several N atoms within a carbon framework, have been reported to display high ORR activity.^4−9^ In addition, degradation issues are usually less severe in alkaline than in acidic environments, entailing higher catalyst durability.^10,11^

Fe/N/C catalysts are synthesized by a thermal treatment under inert or reactive (NH_3_) atmospheres of a physical mixture of Fe, N, and C precursors at temperatures between ca. 700 and 1100 °C.^12,13^ During the thermal treatment, the precursors decompose within a low temperature range, the formation of iron particles is observed along with the observation of carbon domains in the medium temperature range, and, finally, the formation of atomically dispersed iron particles is observed at high temperatures.^14^ A successful strategy for obtaining highly active and durable catalysts is the use of high-molecular-weight precursors with a defined porous structure that allows higher temperatures to be reached during the thermal treatment, hence producing more graphitic materials. Covalent triazine frameworks (CTFs) are a kind of N- and C-containing polymers with high surface areas and controlled textural properties. Furthermore, the synthesis of the CTF follows green chemistry principles.^15,16^ Therefore, CTFs are used in several applications including gas storage and separation (CO_2_ capture and H_2_ storage),^17,18^ electronics,^19^ energy storage,^20,21^ heterogeneous catalysis,^22−24^ photocatalysis^25,26^ and electrocatalysis.^27,28^ CTFs can be synthesized by following different approaches: (a) ionothermal synthesis, which is a reaction at high temperatures in the presence of ZnCl_2_, which melts and acts as an ionic liquid dissolving aromatic dinitrile monomers, also catalyzing the trimerization of nitriles into triazines due to its Lewis acid character;^29^ (b) the phosphorus-pentoxide-catalyzed method, where P_2_O_5_ promotes the direct condensation of aromatic primary amide groups into nitriles and subsequently condenses to produce triazine structures;^30^ (c) the Brønsted superacid synthesis method, where Cooper et al.^31^ reported that chlorosulfonic acid catalyzes the trimerization of aromatic nitriles at room temperature under microwave conditions; (d) amidine polycondensation synthesis, a novel mild synthesis method reported by Tan et al.^32^ showing that a condensation reaction between an aldehyde and amidine hydrochloride under the presence of a Schiff base produces amorphous CTFs followed by an improved oxidation strategy, using alcohols, allowing the creation of crystalline structures; and (e) the Friedel–Crafts reaction method, where an amorphous CTF is obtained when cyanuric chloride reacts with aromatic monomers.^33^

**Scheme 1:**
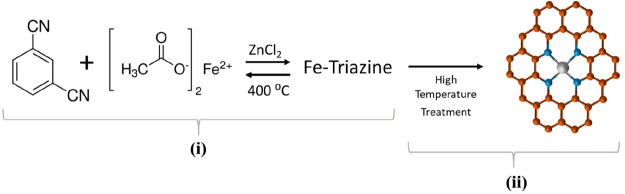
Illustration of the Synthesis of Fe/N/C: (i) Ionothermal Synthesis of Fe-Containing Triazine POP and (ii) Synthesis of Fe/N/C

Because of the chemical flexibility of CTFs, they are promising candidates as a precursor to NPMCs. An important aspect of NPMCs is the actual architecture of the iron–nitrogen active sites, meaning how the atoms are coordinating between them. Iron–nitrogen ensembles can be coordinated in various forms,^34^ such as Fe–N_4_, Fe–N_2+2_, N–Fe–N_2+2_, Fe–N_4+1_, Fe–N_3_, Fe–N_2_, and Fe–N_*x*_–C_4–*x*_. Among all of these configurations, it has been proposed that the low-spin ferrous FeN_4_ and the high-spin N–Fe–N_2+2_ (with a terminal protonated nitrogen) are the most active configurations in acidic media.^35,36^ However, there is still controversy due to recent studies that declare that high-spin ferrous species Fe(III)N_4_C_12_ at the catalyst surface could be the main responsible species.^37^ To the best of our knowledge, such specific studies have not been performed in alkaline media, probably because it has been reported that Fe-free N–C moieties and isolated iron in metallic, carbide, or nitride species also display activity for the ORR in an alkaline system.^38−40^ Therefore, CTFs could be ideal candidates to become the starting core of an inexpensive NPMCs synthesis when looking for a particular iron–nitrogen configuration. The incorporation of iron within the polymerization synthesis of CTFs can lead to the desired electrocatalytic material, hence our recent work based on the novel synthesis of an active NPMC in acidic media and based on the polymerization of a CTF^41^ via an ionothermal high-temperature reaction (Scheme 1). In this work, we have designed two catalysts with high performance in alkaline media, and we have studied the effect of the different heat treatments on a nitrogen/ammonia atmosphere.

## 2 Experimental Section

### 2.1 Synthesis of Fe-Containing Porous Organic Triazine and Fe/N/C Catalysts

The starting materials, 1,3-dicyanobenzene (1,3-DCB), zinc chloride, and iron(II) acetate (all purchased from Aldrich), were mixed in a glovebox in 1:1 DCB/ZnCl_2_ and 0.16 Fe(OCH_3_)_2_/DCB molar ratios. The mixture was ground, placed into a Pyrex vial, and sealed under vacuum. The vial was heated from room temperature (r.t.) to 400 °C at 3 °C/min, kept at 400 °C for 46 h, and cooled to r.t. A black monolith (poly-1,3-DCB) was obtained and ball-milled in a planetary ball mill for 60 min. The recovered solid was thermally treated under a temperature program consisting of a heating ramp from r.t. to 900 °C at 20 °C/min, dwelling at 900 °C for 30 min under NH_3_/N_2_ flows of 28.1 and 24.2 mL/min, respectively, and cooling to r.t. under a N_2_ atmosphere. The catalyst obtained is referred to as 1HT-1,3DCB. To remove the unstable Fe phases, we subjected 1HT-1,3DCB to acid leaching in 0.5 M H_2_SO_4_ at 60 °C for 4 h and washed it with Millipore Milli-Q H_2_O until the pH of the water obtained was ca. 6. Finally, the material was thermally treated following the thermal treatment protocol previously defined. The catalyst obtained is labeled 2HT-1,3DCB.

### 2.2 Characterization

X-ray diffractograms were obtained using an X’Pert Pro PANalytical diffractometer in Bragg–Brentano reflection geometry with Cu K_α_ radiation (λ = 1.5418 Å). C, H, and N contents were measured using a LECO CHNS-932 elemental analyzer.

The textural properties were evaluated using a Micromeritics ASAP 2000 apparatus. Adsorption/desorption nitrogen isotherms within a relative pressure range of *P*/*P*_0_ = 0.05 to 0.30 were selected to evaluate the surface area. A certain volume of gas was absorbed to the surface at −196 °C (nitrogen boiling point) of the sample, which, later on, was degassed at 140 °C under vacuum conditions for 24 h.

Transmission electron microscopy (TEM) images were collected with a 200 kV field-emission gun transmission electron microscope (JEOL 2100F) equipped with an EDX spectrometer Oxford INCA Energy 2000 system. We prepared TEM specimens by dropping the solution of the sample in ethanol on a lacey carbon TEM grid.

X-ray absorption spectroscopy (XAS) measurements were performed on the B18 beamline at the Diamond Light Source UK synchrotron facility.^42^ Spectra were recorded at the Fe K-edge (*E* ≈ 7120 eV). We collected data in fluorescence mode because the spectra showed a small edge jump in transmission signal. Pellets were prepared by mixing <10 mg of sample with cellulose. XAS data were then collected with three repetitions of 3 min (total of ∼10 min) that were then averaged to obtain an improved signal-to-noise ratio. The collected XAS spectra were aligned in energy and normalized to unity edge jump using the Athena software from the Demeter package.^43^ The χ(*k*) Extended X-ray absorption fine structure (EXAFS) signals were also extracted using the same program. The Fourier transforms (FTs) of the EXAFS spectra were obtained by transforming the *k*^2^χ(*k*) functions in the (2–14) Å^–1^ range.

X-ray photoelectron spectra have been collected using a VG Escalab 200 R apparatus using pass energy of 50 eV and a Mg Kα X-ray source. The kinetic energies of the photoelectrons were measured with a hemispherical electron analyzer working in the constant-pass energy mode. A background pressure of 3 × 10^–8^ mbar was kept in the analysis chamber below during the spectra recording. A minimum of 250 scans were collected in increments of 0.1 eV with dwell times of 50 ms to enhance the signal-to-noise ratio. The positions of the photoelectronic peaks under study are referred to the C 1s peak at 284.6 eV.

*Catalytic Performance for the Oxygen Reduction Reaction*: The activity and selectivity for the ORR were assessed in an alkaline electrolyte using an Autolab PGSTAT302N potentiostat/galvanostat connected to a rotating disk electrode (RDE). The working electrode was a glassy carbon disk with a geometric area of 0.196 cm^2^. Metrohm Ag/AgCl KCl (satd) and gold wire were used as the reference and counter electrodes, respectively. For the electrochemical measurements, the catalyst under study was deposited, as an ink, onto the glassy carbon RDE to a catalyst loading of 0.4 mg_cat_cm^–2^. The ink was prepared as follows. 4 mg of catalyst were dispersed on 780 μL of Millipore Milli-Q water, 200 μL of isopropyl alcohol, and 20 μL of 5 wt % Nafion. This mixture was dispersed in an ultrasonic bath for at least 30 min. The ORR activity was measured by recording cyclic voltammograms (CVs) between 0.0 and 1.2 V vs reversible hydrogen electrode (RHE) in O_2_-saturated 0.1 M KOH electrolytes at 10 mV s^–1^ and different rotation rates. In this work, potentials are reported versus the RHE. The potentials recorded were corrected by measuring the electrical impedance spectroscopy (EIS) at open voltage, concluding in a resistance value of 42 Ω, following the equation
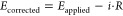
1The durability of the catalysts has been tested by conducting an accelerated stress test (AST) under ORR conditions. Typically, the catalyst under study was loaded onto an RDE (final loading 0.4 mg·cm^–2^_geom_) and subjected to 5000 consecutive cycles between 0.4 and 1.0 V vs RHE at 1600 rpm with a scan rate of 50 mV s^–1^ in an O_2_-saturated electrolyte. To assess the evolution of the catalytic performance, we collected CVs every 500 cycles under the same conditions at 10 mV s^–1^.

## 3 Results and Discussion

### 3.1 Physicochemical Characterization of the Catalysts

The C, H, and N contents and the specific surface areas of 1HT-1,3DCB and 2HT-1,3DCB are shown in Table 1. As shown, the relative content of carbon in 2HT-1,3DCB is significantly higher than that in 1HT-1,3DCB. As discussed as follows, this is because 1HT-1,3DCB contains a significant fraction of iron- and zinc-containing phases that are removed during the acid leaching and second pyrolysis treatment.

**Table 1:** Elemental Analysis and BET Surface of the Catalyst Obtained

	weight content (%)				
catalyst	C	N	H	N/C atomic ratio	specific surface area (micropore/external surface) (m^2^·g^–1^)
1HT-1,3DCB	73.06	1.68	0.66	0.023	374 (168/206)
2HT-1,3DCB	88.42	1.68	0.92	0.019	537 (253/284)

The specific surface areas of the catalysts have been determined from the N_2_ adsorption–desorption isotherms using the Brunauer–Emmett–Teller (BET) method; see Table 1. The BET area of 2HT-1,3DCB, 537 m^2^g^–1^, is significantly higher than that of 1HT-1,3DCB, 374 m^2^g^–1^. However, the relative micropore/external surface areas are similar in both catalysts, as shown in Table 1. This observation suggests that porosity is generated during the thermal treatment, and the higher porosity of 2HT-1,3DCB accounts for the fact that this sample has been subjected to two pyrolysis steps. Note that the surface area of poly-1,3DCB, the monolith obtained after 1,3-dicyanobenzene polymerization at 400 °C under vacuum, is very low, ∼5 m^2^g^–1^.

**Figure 1:**
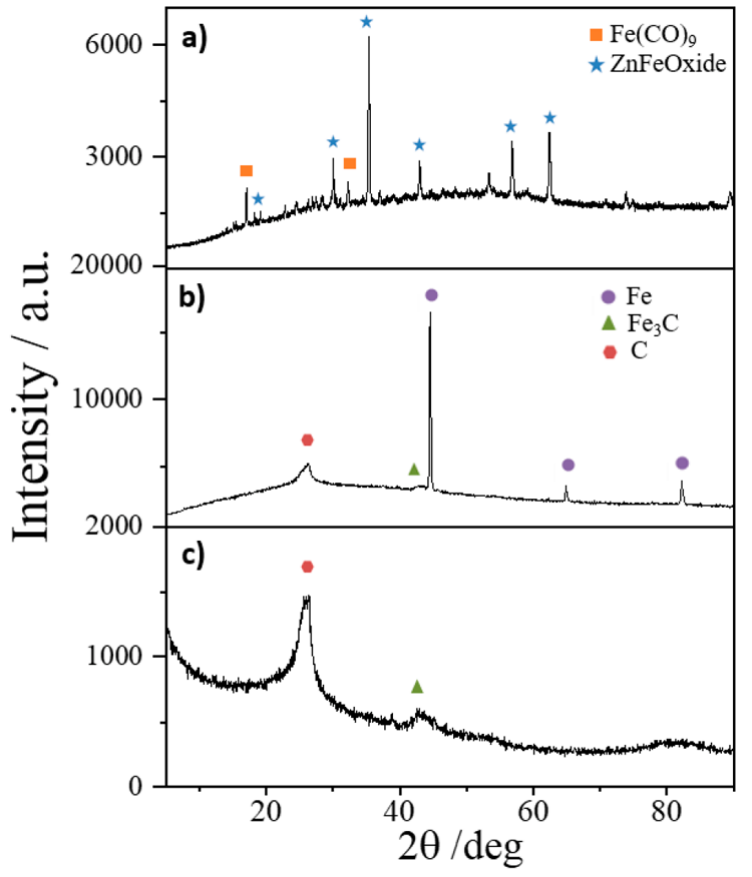
XRD patterns of (a) poly-1,3DCB, (b) 1HT-1,3DCB, and (c) 2HT-1,3DCB catalysts.

Figure 1 shows the X-ray diffraction (XRD) patterns of poly-1,3DCB, 1HT-1,3DCB, and 2HT-1,3DCB. The diffractogram for poly-1,3DCB shows sharp reflections corresponding to Fe_3_O_4_, ZnCl_2_, and Fe_2_(CO)_9_ phases. After the first pyrolysis in NH_3_/N_2_, a strong transformation of the phases is observed, and the diffractogram of 1H-1,3DCB shows reflections for metallic Fe, Fe_3_C, and graphitic carbon. The observation of reduced iron particles (metallic iron and Fe_3_C) and the absence of features for oxidized iron species in the diffractogram of 1HT-1,3-DCB are indicative of the reductive nature of the atmosphere during the pyrolysis step. The diffractogram of 2HT-1,3DCB shows very weak reflections for graphitic carbon and Fe_3_C. This result indicates that metallic iron particles are completely removed during the acid leaching and the second pyrolysis. Despite the fact that the main fraction of Fe_3_C is removed during this treatment, a minority fraction of Fe_3_C particles still remains in 2HT-1,3DCB after acid leaching, as deduced by the very weak set of reflections at 2θ values of ca. 43.5°. The presence of iron carbide particles in the acid-leached sample accounts for the fact that they were wrapped within several graphite layers, therefore preventing their dissolution during acid leaching. In fact, previous studies clearly showed that Fe_3_C dissolution in acid (either acid leaching or the ORR) is a slow process.^44^ To quantify the relative amount of iron carbides in each catalyst, we normalized the area of the diffraction peak for iron carbides (at 2θ ca. 43.5°) with respect to the peak for graphitic carbon (2θ ca. 25.5°) in each sample; see Figure S1 in the Supporting Information. We obtained values of 0.24 and 0.13 for samples 1HT-1,3-DCB and 2HT-1,3-DCB, indicating a decrease in the relative fraction of iron carbides in the latter sample.

**Figure 2:**
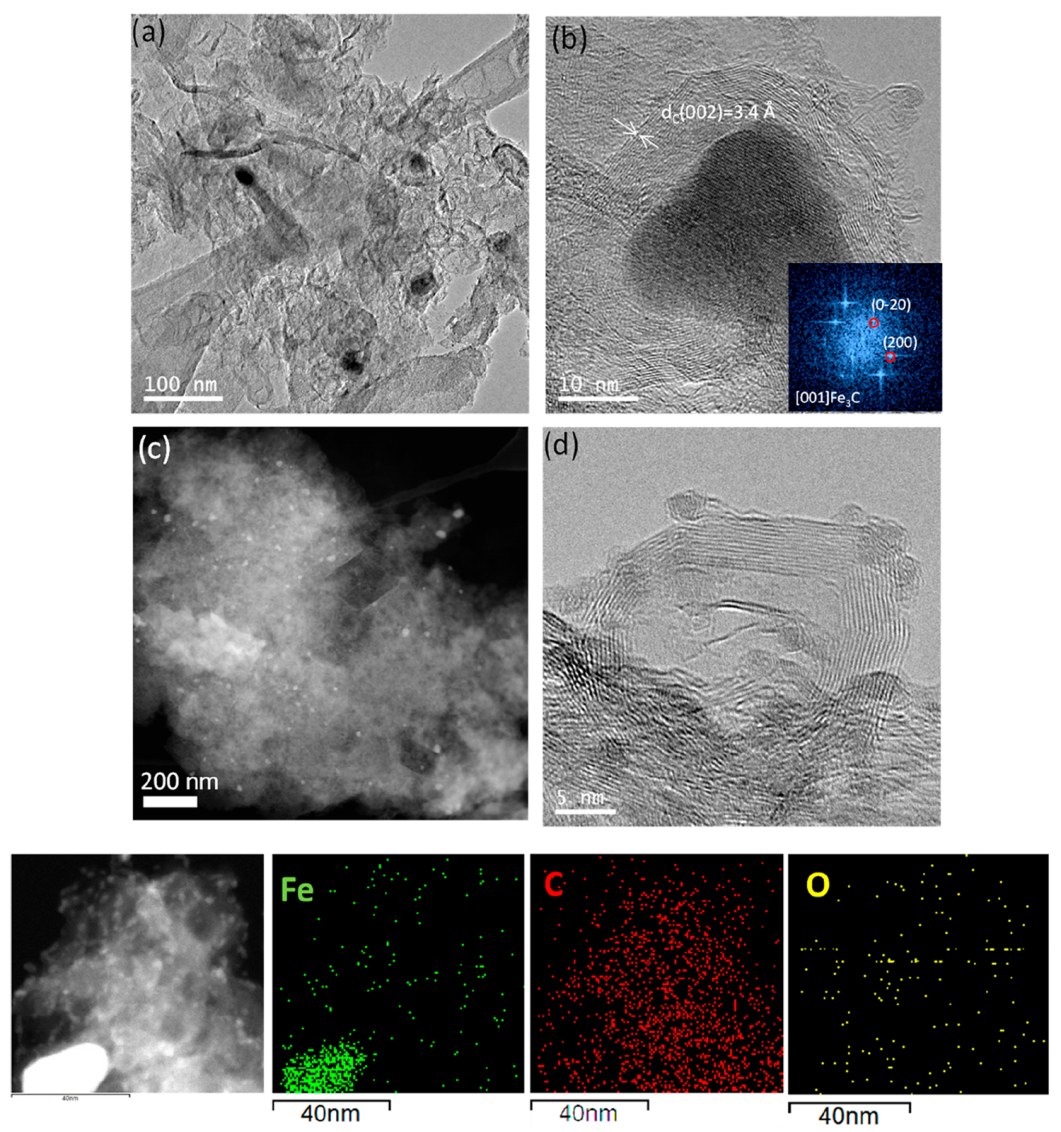
Representative TEM and STEM images of 1HT-1,3DCB catalyst. (a) TEM image of iron particles dispersed onto a carbon matrix. (b) Magnification of an Fe_3_C particle wrapped within several graphite layers and FFT of the iron particle. STEM image showing (c) the homogeneous distribution of iron particles (bright spots) in the catalyst and (d) nanosized iron oxide particles. Bottom panel: Representative STEM image of 1HT-1,3DCB and elemental mapping showing the distribution of Fe, C, and O atoms.

Figure 2 shows representative TEM micrographs of 1HT-1,3DCB. As shown in Figure 2a, 1HT-1,3DCB displays a carbon matrix containing isolated and encapsulated Fe-rich particles. Some of these particles, of around 10–20 nm, are encapsulated within several layers (15–20 layers) of carbon (Figure 2b). The inset to Figure 2b shows the fast Fourier transform (FFT) of the TEM image of one of such particles that can be indexed in the [001] zone axis of the cohenite Fe_3_C structure. The scanning transmission electron microscopy–high-angle annular dark-field (STEM-HAADF) micrograph in Figure 2c reveals the presence of such particles homogeneously dispersed over the carbon matrix. The presence of isolated iron metallic particles of ∼10 nm along with nanosized particles of iron oxides in 1HT-1,3DCB has been confirmed by the TEM analysis; see Figure 2d.

**Figure 3:**
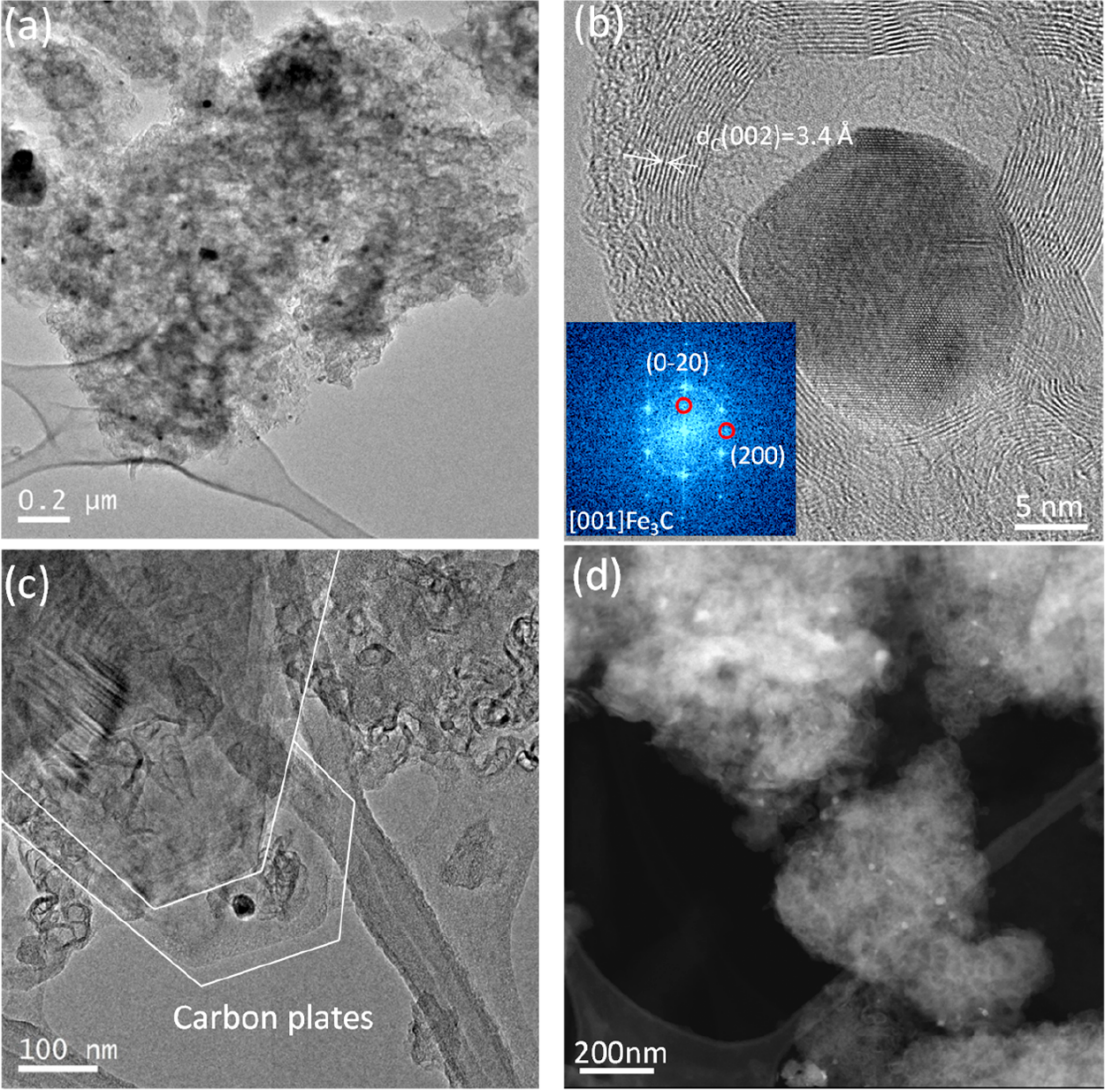
TEM and STEM images of 2HT-1,3DCB. (a) TEM image showing the presence of a small fraction of iron particles on the carbon matrix. (b) High-resolution TEM image showing an Fe_3_C particle (as demonstrated by the FFT of the image, see inset) wrapped within graphite layers. (c) Presence of carbon plates is shown. (d) STEM image showing the presence of a small fraction of iron particles

Figure 3 shows representative TEM and STEM images of 2HT-1,3DCB. The images reveal that the content of iron particles in 2HT-1,3DCB is significantly lower than that in 1HT-1,3DCB. This observation, which is in line with the XRD results, indicates the successful removal of most iron phases during the acid leaching and second pyrolysis. However, the removal of iron particles in not complete (see Figure 3a,d), and a small fraction of Fe_3_C particles encapsulated into several layers of graphitic carbon, as deduced from the FFT images in Figure 3b, can be observed in 2HT-1,3DCB. This observation is in good agreement with the XRD data. TEM images also reveal the morphology of the carbon matrix after the leaching process and that several parts of the carbon matrix transformed, forming carbon plates (Figure 3c).

**Figure 4:**
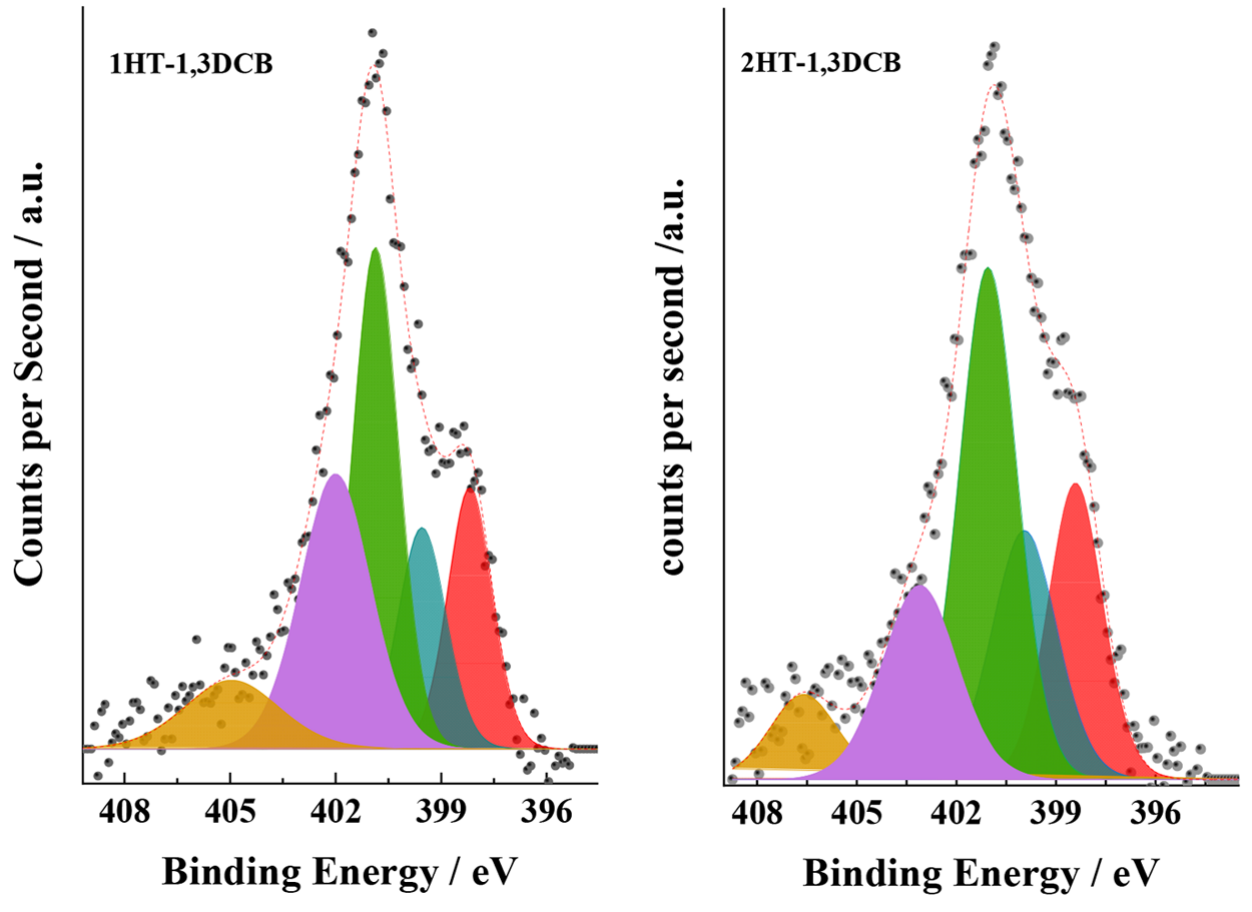
N 1s core-level regions of 1HT-1,3DCB and 2HT-1,3DCB showing the presence of the N-containing species in the catalysts, namely, pyridinic-N (red curve), FeN_*x*_ (blue curve), pyrrolic-N (green curve), N-graphitic (purple curve), and N-oxide (brown curve).

The surface composition of 1HT-1,3DCB and 2HT-1,3DCB was analyzed by X-ray photoelectron spectroscopy (XPS) by recording the N 1s (Figure 4), C 1s (Figure S2), and Fe 2p (Figure S3) core-level regions. The relative surface contents of C, N, and Fe atoms in the catalysts were calculated from the integration of the C 1s, N 1s, and Fe 2p_3/2_ core-level peaks using the corresponding sensitive atomic factors.^45^ As deduced from the evolution of the C/Fe and N/Fe atomic ratios, the content of Fe decreases after the acid leaching and second pyrolysis. (See also that the intensity of the Fe 2p spectra decreases after acid leaching and the second thermal treatment (Figure S3).) The relative C/N content also increases, indicating that N atoms from the surface are also removed during the second treatment. The nature of the N species at the surface of 1HT-1,3DCB and 2HT-1,3DCB catalysts was analyzed by XPS. The N 1s core-level spectra of both catalysts were deconvoluted into five components at ca. 398.4, 399.2, 400.7, 402.6, and 405.6 eV, which, in agreement with previous references, can be ascribed to pyridinic N, N coordinated to Fe (FeN_*x*_ ensembles), pyrrolic N, graphitic and/or N quaternary, and N oxidized, respectively.^40,46^ The fraction of the N-containing species in each catalyst is reported in Table 2. As shown, the fraction of FeN_*x*_ species in 2HT-1,3DCB is higher than that in 1HT-1,3DCB, a feature that has been observed in previous reports and sustains the idea that FeN_*x*_ sites are formed during the thermal treatment at high temperature.^14^ In both catalysts, pyrrolic N is the predominant N-containing species, although the fraction of pyridinic nitrogen increases after the second thermal treatment.

**Table 2:** Fe, N, and C Surface Atomic Ratios and Fraction of N Species Obtained by XPS

	atomic ratios			nitrogen species (%)				
catalyst	C/N	C/O	N/Fe	pyridinic	FeNx	pyrrolic	quaternary	oxide
1HT-1,3DCB	44	17	15	17	14	34	22	13
2HT-1,3DCB	52	13	17	19	21	35	15	10

It is well known that Fe/N/C contains different types of sites (N–Fe ensembles with different coordinations, geometries, spin states, etc.) and that the actual ORR activity of the catalyst depends on the nature of such phases. For instance, the most active sites for the ORR are FeN_*x*_ ensembles, especially FeN_4_ moieties hosted at edge sites. Pyridinic N promotes the four-electron pathway of a direct reduction of O_2_ to H_2_O, whereas N-graphitic is claimed to promote the two-electron pathway of O_2_ to H_2_O_2_.^40,47−49^

The nature of the iron species in 1HT-1,3DCB and 2HT-1,3DCB was also analyzed by XPS (Figure S3). The spectra of both samples display a low intense peak at ca. 710 eV, which is characteristic of oxidized iron species. The observation of this peak in the spectra of similar Fe/N/C catalysts has been ascribed to Fe^2+^ species in FeN_*x*_ moieties, but this binding energy is also characteristic of Fe^2+^ or Fe^3+^ species in iron oxides. However, the photoelectronic spectra of iron ion oxides display a broad shakeup satellite peak at higher binding energies than that of the main photoelectronic peak. The Fe 2p core-level spectra of 1HT-1,3DCB and 2HT-1,3DCB catalysts fail to display shakeup peaks, suggesting that the Fe species in both catalysts are Fe atoms coordinated to N atoms. However, the low intensity of the Fe peaks, due to their small content, cannot rule out the presence of Fe oxides.

**Figure 5:**
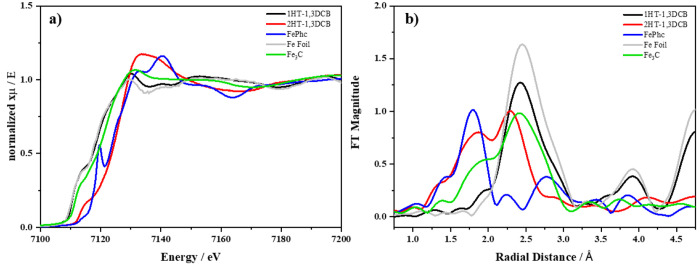
(a) Fe K-edge XANES and (b) Fourier transform of the EXAFS signals of 1HT-1,3DCB (black line) and 2HT-1,3DCB (red line) and for the standards, Fe-phthalocyanine (blue line), Fe_3_C (green line), and iron foil (gray line).

The oxidation state and environment of the iron species in 1HT-1,3DCB and 2HT-1,3DCB were further studied with XAS. The Fe K-edge energy and X-ray absorption near edge structure (XANES) spectral features for 1HT-1,3DCB (Figure 5a) appear to be similar to the XANES spectrum of the metallic iron foil (used as standard), indicating that the Fe atoms in 1HT-1,3DCB are mainly present as metallic Fe. This result, in good agreement with XRD and TEM results, reveals that 1HT-1,3DCB is mainly composed of metallic Fe and Fe_3_C. The XANES spectrum of 2HT-1,3DCB shows a shift of the edge toward higher energies, which is very close to that of Fe-phthalocyanine, indicating the preponderance of Fe atoms in the 2+ oxidation state. Fe atoms in 2HT-1,3DCB adopt a different geometry than that of Fe in Fe-phthalocyanine. In the latter, a clear prepeak is observed that does not appear in the spectra of 2HT-1,3DCB. This prepeak is characteristic of Fe in the square-planar environment of FeN_4_ ensembles.^50^ As we have previously reported,^41^ the different pre-edge feature in 2HT-1,3DCB indicates that the geometry of the FeN_*x*_ moieties is not the same as that in Fe-phthalocyanine. The different geometry can be related to the bending of the FeN_*x*_ moieties or to the occupation of some of the axial empty positions of the square planar FeN_4_ moieties.^51^

Figure S4 and Figure 5b show the *k*-space EXAFS and the phase-corrected FT-EXAFS spectra of the Fe K-edge, respectively. The FT-EXAFS for 1HT-1,3DCB is dominated by a peak at ca. 2.4 Å, which corresponds to Fe–Fe bond distances, in good agreement with the presence of Fe_3_C and Fe^0^. The spectrum of 2HT-1,3DCB shows two main peaks at ca. 1.8 and 2.4 Å, ascribed to Fe–N and Fe–Fe distances, respectively, indicative of the presence of FeN_*x*_ and Fe_3_C, respectively. This result, which is in line with the XPS results, reveals that the acid leaching and second thermal treatment lead to the removal of metallic iron phases (including Fe_3_C species) and to the formation of further FeN_*x*_ ensembles, resulting in the preponderance of the latter iron species in 2HT-1,3DCB.

### 3.2 Oxygen Reduction Activity in an Alkaline Electrolyte

**Figure 6:**
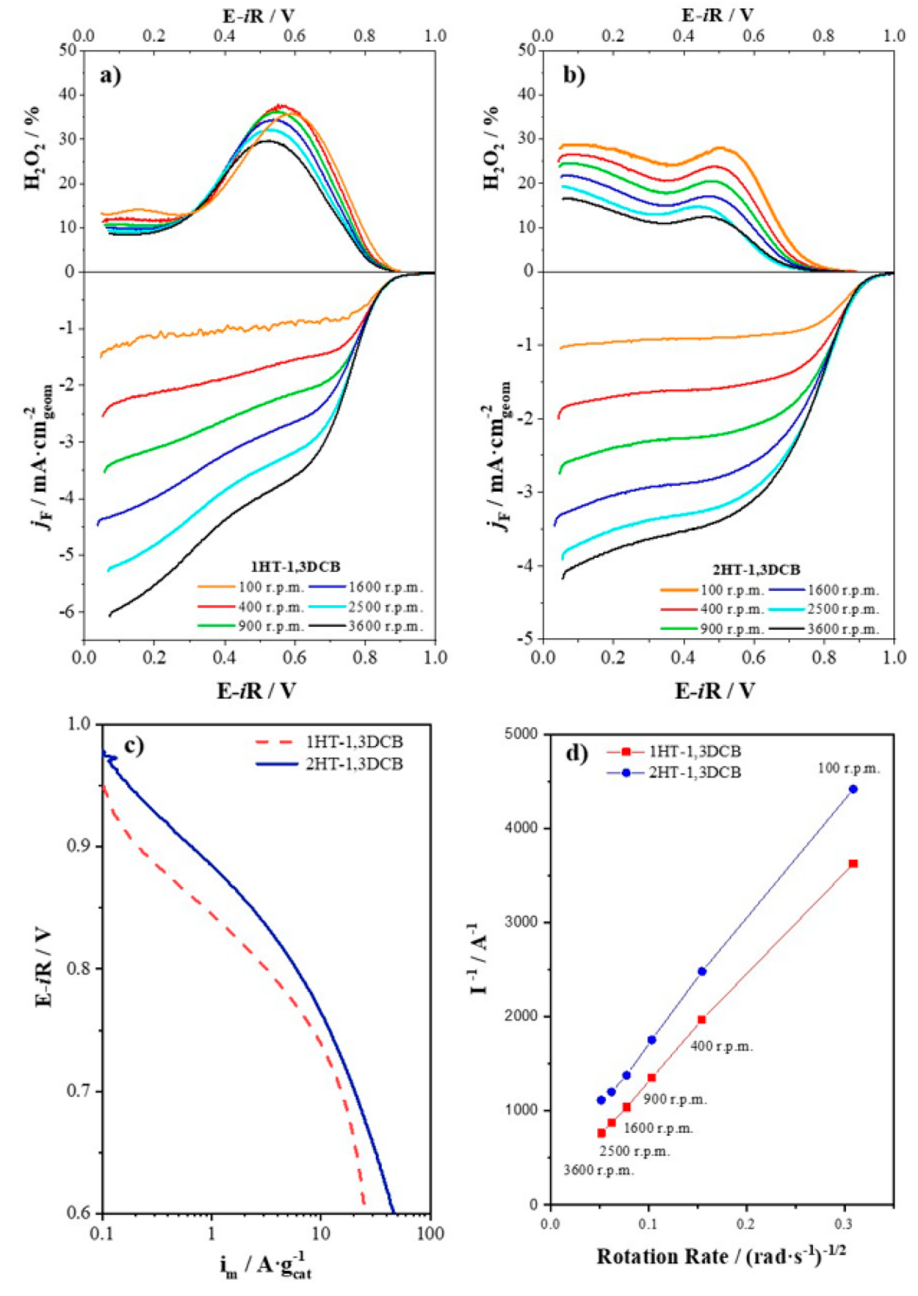
Polarization curves for (a) 1HT-1,3DCB and (b) 2HT-1,3DCB recorded in O_2_-saturated 0.1 M KOH electrolyte in a positive-going scan at 10 mV s^–1^ at different rotation rates. The upper panels show the production of H_2_O_2_ with each catalyst. (c) Tafel plots (*E* vs log *i*_m_) from the ORR polarization curves at 1600 rpm. (d) Koutecky–Levich plots for 1HT-1,3DCB and 2HT-1,3DCB at *E* = 0.2 V.

The electrocatalytic performance of 1HT-1,3DCB and 2HT-1,3DCB for the ORR was measured in an alkaline electrolyte. The RDE thin-film technique is used for the measurements.^52^Figure 6 illustrates the ORR polarization curves for 1HT-1,3DCB and 2HT-1,3DCB in O_2_-saturated 0.1 M KOH recorded at different rotation rates. Both catalysts show activity for the ORR, and they present distinct ORR activities. Thus 1HT-1,3DCB displays only moderate ORR activity, with *E*_onset_ at 0.89 V. Noticeably a plateau current is not reached, probably indicating a low fraction of active sites in the catalyst or a high production of hydrogen peroxide; see as follows. On the contrary, 2HT-1,3DCB displays a superior performance of ORR, with *E*_onset_ at 0.93 V (see Table 3 and the discussion that follows), and reaches a better-defined limiting current. The activity of 2HT-1,3DCB is, in fact, comparable to that of state-of-the-art NPMCs in alkaline media.^13^

**Table 3:** ORR Onset Potential, Half-Wave Potential, and Mass Activities for the Catalysts under Study

catalyst	catalyst loading in RDE (μg/cm^2^)	*E*_onset_ (V)	*E*_1/2_ (V)	*i*_m_ (A/g) at 0.9 V	*i*_m_ (A/g) at 0.8 V	*i*_m_ (A/g) at 0.7 V	ref
1HT-1,3DCB	400	0.89	0.71	0.2	3.2	15.4	this work
2HT-1,3DCB	400	0.93	0.77	0.7	6.0	20.4	this work
Fe-N/AB	637	0.92	0.81				(54)
Fe-N/MWCNT	637	0.97	0.84				(54)
C-Fe-ZIF-900-2.53	500	0.95	0.82				(55)
Fe-NMG	600	0.96	0.83				(56)

The mass activities for the catalysts under study (see Figure 6c) have been calculated from the pure kinetic currents derived from the Koutecky–Levich (K–L) equation
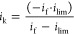
2where *i*_k_ is the kinetic current, which is negative for reduction reactions, and *i*_lim_ is the limiting current. The ORR mass activity is determined by the following equation
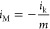
3where *m* is the loading of the catalyst in the electrode. In Figure S5, it is shown that 1HT-1,3DCB and 2HT-1,3DCB record similar mass activities at low overpotentials. However, at higher overpotentials, that is, at potentials less positive than 0.8 V, the mass activities diverge, and 2HT-1,3DCB displays higher mass activities.

The ORR can proceed via the four-electron or the two-electron pathways, producing H_2_O or H_2_O_2_, respectively. The production of hydrogen peroxide during the ORR is not desirable because it implies a lower efficiency (two electrons vs four electrons exchanged per O_2_ molecule) and because the presence of H_2_O_2_ can accelerate the corrosion of the catalyst. The production of H_2_O_2_ during the ORR with 1HT-1,3DCB and 2HT-1,3DCB was assessed using a rotating ring disk electrode (RRDE) with a Pt ring; see Figure 6. The potential of the ring electrode is set at 1.2 V vs RHE to ensure the oxidation of the H_2_O_2_ produced during the ORR. The determination of the current associated with the oxidation of H_2_O_2_ into O_2_ (*i*_R_) allows one to quantify the fraction of H_2_O_2_ formed during the ORR using eq 4.^53^ In eq 4, *i*_R_ and *i*_D_ are the ring and disk faradaic currents, respectively, and *N* is the ring collection efficiency (in this case, 38%). RRDE tests show a hydrogen peroxide production of ca. 35 and 17% for 1HT-1,3DCB and 2HT-1,3DCB catalysts, respectively; see Figure 3b.
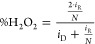
4The following descriptors are used to assess the ORR activity of the electrocatalysts: (i) the onset potential (*E*_onset_), which is the potential when a current density of 0.1 mA/cm^2^ is achieved; (ii) the half-wave potential (*E*_1/2_), which is the potential at half of the limiting current; and (iii) the mass activities (*i*_m_) at 0.9, 0.8, and 0.7 V. (See Figure 6 and Table 3.) As previously stated, both catalysts display high ORR activity in an alkaline electrolyte, especially 2HT-1,3DCB, displaying values that compare well with the best catalyst reported in the literature, with *E*_1/2_ between 0.80 and 0.84 V for state-of-the-art ORR catalysts in an alkaline electrolyte, namely, Fe-N/AB and Fe-N/MWCNT,^54^ C-Fe-ZIF-900-2.53,^55^ and Fe-NMG.^56^ (See Table 3.) The beneficial effect of the second pyrolysis treatment is clear by comparing the ORR performance of 1HT1-3DCB and 2HT-1,3DCB. The latter catalyst displays superior ORR performance in terms of *E*_onset_, *E*_1/2_, and *i*_m_ when compared at the same potentials. In addition, 2HT-1,3DCB displays a selectivity for the four-electron pathway, that is, a lower formation of H_2_O_2_ than 1HT-1,3DCB. This superior performance can be ascribed to the combination of a higher content of FeN_*x*_ ensembles, confirming the idea that FeN_*x*_ sites are the most active sites for the ORR,^11^ and to the higher BET area of the catalyst subjected to a second pyrolysis.

As previously stated, the ORR polarization curves for 1HT-1,3DCB do not reach proper plateau currents. In addition, both catalysts fail to reach the theoretical limiting current value expected for a four-electron process defined by the Levich equation at any rotation rate. (See Figure 6a,b.) According to the K–L equation, eq 5, the number of exchanged electrons can be calculated from the current densities obtained at different rotation rates. By plotting the reciprocal of the current against the reciprocal of the rotation rate square root, straight lines are obtained, and the slope of the lines can be used to calculate the number of exchanged electrons.
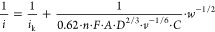
5where *F* is the Faraday constant, *D* is the diffusion coefficient of O_2_ in 0.1 M KOH, which is equivalent to 1.93 × 10^–5^cm^2^·s^–1^, *v* is the kinematic viscosity of the electrolyte, which is equivalent to 1.09 × 10^–2^ cm^2^·s^–1^, and *C* is the saturation concentration of O_2_ in 0.1 M KOH, which is equivalent to 1.96 × 10^–6^ mol·cm^–3^ due to the fact that the experiments were done at high altitude (∼700 m above sea level), where the O_2_ pressure is 0.89 atm.

From Figure 6d, a total number of exchanged electrons of 3.2 e^–^ and 3.7 e^–^ was obtained at 0.2 V for 1HT-1,3DCB and 2HT-1,3DCB, respectively. These values compare well with the average values deduced from the H_2_O_2_ production recorded in the RRDE experiments of 3.3 e^–^ and 3.6 e^–^ for 1HT-1,3DCB and 2HT-1,3DCB, respectively.

The ORR activity results previously shown, that is, a higher ORR activity and a lower production of H_2_O_2_ recorded with 2HT-1,3DCB, can be related to the different iron phases in both catalysts. As shown by XRD and TEM, the acid leaching removes the iron phases that are not stable in acid media, especially metallic iron and iron carbides. Although it is admitted that such iron species can be active for the ORR in an alkaline electrolyte,^34,38,57^ they promote the two-electron reaction pathway, that is, the production of H_2_O_2_. Because 1HT-1,3DCB displays a high fraction of iron particles (mostly graphite wrapped Fe_3_C), it displays high ORR activity, but via the two-electron pathway. After acid leaching, the total amount of iron in the catalyst decreases, especially due to the dissolution of Fe_3_C. Additionally, as shown by XPS, the second thermal treatment results in a higher density of FeN_*x*_ sites. This is confirmed by the Fe K-edge XAS analysis, which reveals a higher fraction of FeN_*x*_ species in 2HT-1,3DCB than in 1HT-1,3DCB. This transformation, the removal of iron particles, and formation of more FeN_*x*_ ensembles result in a higher ORR activity and a lower production of H_2_O_2_.

Finally, we have carried out an AST under ORR conditions to evaluate the durability and stability of 1HT-1,3DCB and 2HT-1,3DCB. Figure S5 shows the polarization curves recorded after every 500 cycles during an AST consisting of 5000 cycles in O_2_-saturated HClO_4_ at 10 mV s^–1^ and 1600 rpm. As shown, the ORR activity of both catalysts decreases with the number of cycles, but the activity loss is more pronounced with 1HT-1,3DCB, decreasing during the whole duration of the experiment. On the contrary, the ORR activity of 2HT-1,3DCB stabilizes after 3000 cycles. The half-wave potential (*E*_1/2_) of 1HT-1,3DCB shifts to less positive values by 51 mV after 4000 cycles and by 118 mV after 5000 cycles. In addition, the limiting current recorded with 1HT-1,3DCB decreases during the 5000 cycles, probably due to a strong loss of active sites. The higher stability of 2HT-1,3DCB during the ORR results in a moderate shifting of the *E*_1/2_ of only 33 mV after 5000 cycles, which is in line with previous reported works.^58−60^ The higher durability of 2HT-1,3DCB probably accounts for the lack of unstable phases in this catalyst after the removal of the soluble phases during acid leaching, typically metallic iron and iron carbide clusters.

## 4 Conclusions

Two Fe/N/C catalysts have been synthesized using a N-containing polymer obtained by the polymerization of 1,3-dicyanobenzene (poly-1,3-DCB) under ionothermal conditions. The first catalyst, 1HT-1,3DCB, was obtained by the thermal treatment of poly-1,3-DCB in the presence of an iron precursor. The second catalyst, 2HT-1,3-DCB, was obtained by acid leaching of 1HT-1,3DCB followed by a thermal treatment. Both catalysts display high ORR activity in an alkaline electrolyte. The characterization results, including XRD, TEM, XPS, and Fe K-edge XAS, reveal that 1HT-1,3DCB contains iron particles, mostly graphite-wrapped Fe_3_C along with a minor fraction of FeN_*x*_ ensembles. However, after acid leaching and the second thermal treatment, FeN_*x*_ ensembles are the main iron-containing species in the catalyst, with only a small fraction of Fe_3_C particles. The ORR activity of both catalysts is affected by the nature of the iron species. Thus the higher fraction of FeN_*x*_ ensembles after acid leaching results in a higher ORR activity and a lower H_2_O_2_ production than 1HT-1,3DCB. In addition, the removal of iron particles (metallic iron and Fe_3_C) by acid leaching results in more stable catalysts during the ORR in an alkaline electrolyte. The results presented in this Article show that the presence of iron particles in Fe/N/C catalysts compromises the ORR durability in an alkaline electrolyte and that removal of such iron particles is recommended for the design of robust, durable ORR catalysts in an alkaline electrolyte.

## Supporting Information Available

X-ray diffractograms, XPS of C 1s and Fe 2p, K-space EXAFS of catalysts and standards, and accelerated stress tests (PDF)

## References

[ref1] Ramaswamy N.; Mukerjee S. Alkaline Anion-Exchange Membrane Fuel Cells: Challenges in Electrocatalysis and Interfacial Charge Transfer. Chem. Rev. 2019, 119 (23), 11945–11979. 10.1021/acs.chemrev.9b00157. 31702901

[ref2] Santori P. G.; Speck F. D.; Li J.; Zitolo A.; Jia Q.; Mukerjee S.; Cherevko S.; Jaouen F. Effect of Pyrolysis Atmosphere and Electrolyte PH on the Oxygen Reduction Activity, Stability and Spectroscopic Signature of FeN x Moieties in Fe-N-C Catalysts. J. Electrochem. Soc. 2019, 166 (7), F3311–F3320. 10.1149/2.0371907jes.

[ref3] Domínguez C.; Pérez-Alonso F. J.; Gómez de la Fuente J. L.; Al-Thabaiti S. A.; Basahel S. N.; Alyoubi A. O.; Alshehri A. A.; Peña M. A.; Rojas S. Influence of the Electrolyte for the Oxygen Reduction Reaction with Fe/N/C and Fe/N/CNT Electrocatalysts. J. Power Sources 2014, 271, 87–96. 10.1016/j.jpowsour.2014.07.173.

[ref4] Proietti E.; Jaouen F.; Lefèvre M.; Larouche N.; Tian J.; Herranz J.; Dodelet J.-P. P. Iron-Based Cathode Catalyst with Enhanced Power Density in Polymer Electrolyte Membrane Fuel Cells. Nat. Commun. 2011, 2 (1), 416 10.1038/ncomms1427. 21811245

[ref5] Wu G.; More K. L.; Xu P.; Wang H. L.; Ferrandon M.; Kropf A. J.; Myers D. J.; Ma S.; Johnston C. M.; Zelenay P. A Carbon-Nanotube-Supported Graphene-Rich Non-Precious Metal Oxygen Reduction Catalyst with Enhanced Performance Durability. Chem. Commun. 2013, 49 (32), 3291–3293. 10.1039/c3cc39121c. 23420477

[ref6] Wu G.; More K. L.; Johnston C. M.; Zelenay P. High-Performance Electrocatalysts for Oxygen Reduction Derived from Polyaniline, Iron, and Cobalt. Science 2011, 332 (6028), 443–447. 10.1126/science.1200832. 21512028

[ref7] Domínguez C.; Pérez-Alonso F. J.; Abdel Salam M.; Al-Thabaiti S. A.; Obaid A. Y.; Alshehri A. A.; Gómez de la Fuente J. L.; Fierro J. L. G.; Rojas S. On the Relationship between N Content, Textural Properties and Catalytic Performance for the Oxygen Reduction Reaction of N/CNT. Appl. Catal., B 2015, 162, 420–429. 10.1016/j.apcatb.2014.07.002.

[ref8] Domínguez C.; Pérez-Alonso F. J.; Abdel Salam M.; Gómez de la Fuente J. L.; Al-Thabaiti S. A.; Basahel S. N.; Peña M. A.; Fierro J. L. G.; Rojas S. Effect of Transition Metal (M: Fe, Co or Mn) for the Oxygen Reduction Reaction with Non-Precious Metal Catalysts in Acid Medium. Int. J. Hydrogen Energy 2014, 39 (10), 5309–5318. 10.1016/j.ijhydene.2013.12.078.

[ref9] Domínguez C.; Pérez-Alonso F. J.; Salam M. A.; Al-Thabaiti S. A.; Peña M. A.; García-García F. J.; Barrio L.; Rojas S. Repercussion of the Carbon Matrix for the Activity and Stability of Fe/N/C Electrocatalysts for the Oxygen Reduction Reaction. Appl. Catal., B 2016, 183, 185–196. 10.1016/j.apcatb.2015.10.043.

[ref10] Kumar K.; Dubau L.; Mermoux M.; Li J.; Zitolo A.; Nelayah J.; Jaouen F.; Maillard F. On the Influence of Oxygen on the Degradation of Fe-N-C Catalysts. Angew. Chem., Int. Ed. 2020, 59 (8), 3235–3243. 10.1002/anie.201912451. 31799800

[ref11] Zagal J. H.; Specchia S.; Atanassov P. Mapping Transition Metal-MN4Macrocyclic Complex Catalysts Performance for the Critical Reactivity Descriptors. Curr. Opin. Electrochem. 2021, 27, 100683 10.1016/j.coelec.2020.100683.

[ref12] Jaouen F.; Jones D.; Coutard N.; Artero V.; Strasser P.; Kucernak A. Toward Platinum Group Metal-Free Catalysts for Hydrogen/Air Proton-Exchange Membrane Fuel Cells. Johnson Matthey Technol. Rev. 2018, 62 (2), 231–255. 10.1595/205651318X696828.

[ref13] Sa Y. J.; Woo J.; Joo S. H. Strategies for Enhancing the Electrocatalytic Activity of M-N/C Catalysts for the Oxygen Reduction Reaction. Top. Catal. 2018, 61 (9–11), 1077–1100. 10.1007/s11244-018-0935-0.

[ref14] Huang Y.; Chen Y.; Xu M.; Asset T.; Tieu P.; Gili A.; Kulkarni D.; De Andrade V.; De Carlo F.; Barnard H. S.; Doran A.; Parkinson D. Y.; Pan X.; Atanassov P.; Zenyuk I. V. Catalysts by Pyrolysis: Direct Observation of Chemical and Morphological Transformations Leading to Transition Metal-Nitrogen-Carbon Materials. Mater. Today 2021, 47, 53–68. 10.1016/j.mattod.2021.02.006.

[ref15] Liu M.; Guo L.; Jin S.; Tan B. Covalent Triazine Frameworks: Synthesis and Applications. J. Mater. Chem. A 2019, 7 (10), 5153–5172. 10.1039/C8TA12442F.

[ref16] Krishnaraj C.; Jena H. S.; Leus K.; Van Der Voort P. Covalent Triazine Frameworks - a Sustainable Perspective. Green Chem. 2020, 22 (4), 1038–1071. 10.1039/C9GC03482J.

[ref17] Hug S.; Stegbauer L.; Oh H.; Hirscher M.; Lotsch B. V. Nitrogen-Rich Covalent Triazine Frameworks as High-Performance Platforms for Selective Carbon Capture and Storage. Chem. Mater. 2015, 27 (23), 8001–8010. 10.1021/acs.chemmater.5b03330.

[ref18] Yu W.; Gu S.; Fu Y.; Xiong S.; Pan C.; Liu Y.; Yu G. Carbazole-Decorated Covalent Triazine Frameworks: Novel Nonmetal Catalysts for Carbon Dioxide Fixation and Oxygen Reduction Reaction. J. Catal. 2018, 362, 1–9. 10.1016/j.jcat.2018.03.021.

[ref19] Zhao X. Novel Porous Materials for Emerging Applications. J. Mater. Chem. 2006, 16 (7), 623–625. 10.1039/b600327n.

[ref20] Liu J.; Lyu P.; Zhang Y.; Nachtigall P.; Xu Y. New Layered Triazine Framework/Exfoliated 2D Polymer with Superior Sodium-Storage Properties. Adv. Mater. 2018, 30 (11), 1705401 10.1002/adma.201705401. 29359817

[ref21] Xu F.; Yang S.; Jiang G.; Ye Q.; Wei B.; Wang H. Fluorinated, Sulfur-Rich, Covalent Triazine Frameworks for Enhanced Confinement of Polysulfides in Lithium-Sulfur Batteries. ACS Appl. Mater. Interfaces 2017, 9 (43), 37731–37738. 10.1021/acsami.7b10991. 28990391

[ref22] Puthiaraj P.; Lee Y.-R.; Zhang S.; Ahn W.-S. Triazine-Based Covalent Organic Polymers: Design, Synthesis and Applications in Heterogeneous Catalysis. J. Mater. Chem. A 2016, 4 (42), 16288–16311. 10.1039/C6TA06089G.

[ref23] Palkovits R.; Antonietti M.; Kuhn P.; Thomas A.; Schüth F. Solid Catalysts for the Selective Low-Temperature Oxidation of Methane to Methanol. Angew. Chem., Int. Ed. 2009, 48 (37), 6909–6912. 10.1002/anie.200902009. 19655358

[ref24] Zhang B.; Wei M.; Mao H.; Pei X.; Alshmimri S. A.; Reimer J. A.; Yaghi O. M. Crystalline Dioxin-Linked Covalent Organic Frameworks from Irreversible Reactions. J. Am. Chem. Soc. 2018, 140 (40), 12715–12719. 10.1021/jacs.8b08374. 30247881

[ref25] Guo L.; Niu Y.; Xu H.; Li Q.; Razzaque S.; Huang Q.; Jin S.; Tan B. Engineering Heteroatoms with Atomic Precision in Donor-Acceptor Covalent Triazine Frameworks to Boost Photocatalytic Hydrogen Production. J. Mater. Chem. A 2018, 6 (40), 19775–19781. 10.1039/C8TA07391K.

[ref26] Xie J.; Shevlin S. A.; Ruan Q.; Moniz S. J. A.; Liu Y.; Liu X.; Li Y.; Lau C. C.; Guo Z. X.; Tang J. Efficient Visible Light-Driven Water Oxidation and Proton Reduction by an Ordered Covalent Triazine-Based Framework. Energy Environ. Sci. 2018, 11 (6), 1617–1624. 10.1039/C7EE02981K.

[ref27] Guo J.; Xu Y.; Jin S.; Chen L.; Kaji T.; Honsho Y.; Addicoat M. A.; Kim J.; Saeki A.; Ihee H.; Seki S.; Irle S.; Hiramoto M.; Gao J.; Jiang D. Conjugated Organic Framework with Three-Dimensionally Ordered Stable Structure and Delocalized π Clouds. Nat. Commun. 2013, 4 (1), 2736 10.1038/ncomms3736. 24220603 PMC3868157

[ref28] Kamiya K.; Kamai R.; Hashimoto K.; Nakanishi S. Platinum-Modified Covalent Triazine Frameworks Hybridized with Carbon Nanoparticles as Methanol-Tolerant Oxygen Reduction Electrocatalysts. Nat. Commun. 2014, 5 (1), 5040 10.1038/ncomms6040. 25242214 PMC4199112

[ref29] Kuhn P.; Antonietti M.; Thomas A. Porous, Covalent Triazine-Based Frameworks Prepared by Ionothermal Synthesis. Angew. Chem., Int. Ed. 2008, 47 (18), 3450–3453. 10.1002/anie.200705710. 18330878

[ref30] Yu S.-Y.; Mahmood J.; Noh H.-J.; Seo J.-M.; Jung S.-M.; Shin S.-H.; Im Y.-K.; Jeon I.-Y.; Baek J.-B. Direct Synthesis of a Covalent Triazine-Based Framework from Aromatic Amides. Angew. Chem., Int. Ed. 2018, 57 (28), 8438–8442. 10.1002/anie.201801128. 29624829

[ref31] Ren S.; Bojdys M. J.; Dawson R.; Laybourn A.; Khimyak Y. Z.; Adams D. J.; Cooper A. I. Porous, Fluorescent, Covalent Triazine-Based Frameworks Via Room-Temperature and Microwave-Assisted Synthesis. Adv. Mater. 2012, 24 (17), 2357–2361. 10.1002/adma.201200751. 22488602

[ref32] Wang K.; Yang L.-M.; Wang X.; Guo L.; Cheng G.; Zhang C.; Jin S.; Tan B.; Cooper A. Covalent Triazine Frameworks via a Low-Temperature Polycondensation Approach. Angew. Chem., Int. Ed. 2017, 56 (45), 14149–14153. 10.1002/anie.201708548. PMC5698698 28926688

[ref33] Puthiaraj P.; Cho S.-M.; Lee Y.-R.; Ahn W.-S. Microporous Covalent Triazine Polymers: Efficient Friedel-Crafts Synthesis and Adsorption/Storage of CO2 and CH4. J. Mater. Chem. A 2015, 3 (13), 6792–6797. 10.1039/C5TA00665A.

[ref34] Asset T.; Atanassov P. Iron-Nitrogen-Carbon Catalysts for Proton Exchange Membrane Fuel Cells. Joule 2020, 4, 33–44. 10.1016/j.joule.2019.12.002.

[ref35] Kramm U. I.; Herranz J.; Larouche N.; Arruda T. M.; Lefèvre M.; Jaouen F.; Bogdanoff P.; Fiechter S.; Abs-Wurmbach I.; Mukerjee S.; Dodelet J. P. Structure of the Catalytic Sites in Fe/N/C-Catalysts for O 2-Reduction in PEM Fuel Cells. Phys. Chem. Chem. Phys. 2012, 14 (33), 11673–11688. 10.1039/c2cp41957b. 22824866 PMC3429934

[ref36] Li J.; Sougrati M. T.; Zitolo A.; Ablett J. M.; Oǧuz I. C.; Mineva T.; Matanovic I.; Atanassov P.; Huang Y.; Zenyuk I.; Di Cicco A.; Kumar K.; Dubau L.; Maillard F.; Dražić G.; Jaouen F. Identification of Durable and Non-Durable FeNx Sites in Fe-N-C Materials for Proton Exchange Membrane Fuel Cells. Nat. Catal. 2021, 4 (1), 10–19. 10.1038/s41929-020-00545-2.

[ref37] Mineva T.; Matanovic I.; Atanassov P.; Sougrati M.-T.; Stievano L.; Clémancey M.; Kochem A.; Latour J.-M.; Jaouen F. Understanding Active Sites in Pyrolyzed Fe-N-C Catalysts for Fuel Cell Cathodes by Bridging Density Functional Theory Calculations and 57 Fe Mössbauer Spectroscopy. ACS Catal. 2019, 9 (10), 9359–9371. 10.1021/acscatal.9b02586.

[ref38] Ahmed M. S.; Begum H.; Kim Y.-B. Iron Nanoparticles Implanted Metal-Organic-Frameworks Based Fe-N-C Catalysts for High-Performance Oxygen Reduction Reaction. J. Power Sources 2020, 451, 227733 10.1016/j.jpowsour.2020.227733.

[ref39] Strickland K.; Miner E.; Jia Q.; Tylus U.; Ramaswamy N.; Liang W.; Sougrati M. T.; Jaouen F.; Mukerjee S. Highly Active Oxygen Reduction Non-Platinum Group Metal Electrocatalyst without Direct Metal-Nitrogen Coordination. Nat. Commun. 2015, 6, 7343 10.1038/ncomms8343. 26059552 PMC4490352

[ref40] Artyushkova K.; Serov A.; Rojas-Carbonell S.; Atanassov P. Chemistry of Multitudinous Active Sites for Oxygen Reduction Reaction in Transition Metal-Nitrogen-Carbon Electrocatalysts. J. Phys. Chem. C 2015, 119 (46), 25917–25928. 10.1021/acs.jpcc.5b07653.

[ref41] García Á.; Retuerto M.; Dominguez C.; Pascual L.; Ferrer P.; Gianolio D.; Serrano A.; Aßmann P.; Sanchez D. G.; Peña M. A.; Rojas S. Fe Doped Porous Triazine as Efficient Electrocatalysts for the Oxygen Reduction Reaction in Acid Electrolyte. Appl. Catal., B 2020, 264, 118507 10.1016/j.apcatb.2019.118507.

[ref42] Dent A. J.; Cibin G.; Ramos S.; Parry S. A.; Gianolio D.; Smith A. D.; Scott S. M.; Varandas L.; Patel S.; Pearson M. R.; Hudson L.; Krumpa N. A.; Marsch A. S.; Robbins P. E. Performance of B18, the Core EXAFS Bending Magnet Beamline at Diamond. J. Phys.: Conf. Ser. 2013, 430, 012023 10.1088/1742-6596/430/1/012023.

[ref43] Ravel B.; Newville M. Athena, Artemis, Hephaestus: Data Analysis for X-Ray Absorption Spectroscopy Using IFEFFIT. J. Synchrotron Radiat. 2005, 12 (Pt 4), 537–541. 10.1107/S0909049505012719. 15968136

[ref44] García Á.; Pascual L.; Ferrer P.; Gianolio D.; Held G.; Grinter D. C.; Peña M. A.; Retuerto M.; Rojas S. Study of the Evolution of FeN C and Fe3C Species in Fe/N/C Catalysts during the Oxygen Reduction Reaction in Acid and Alkaline Electrolyte. J. Power Sources 2021, 490, 229487 10.1016/j.jpowsour.2021.229487.

[ref45] Wagner C. D.; Davis L. E.; Zeller M. V.; Taylor J. A.; Raymond R. H.; Gale L. H. Empirical Atomic Sensitivity Factors for Quantitative Analysis by Electron Spectroscopy for Chemical Analysis. Surf. Interface Anal. 1981, 3 (5), 211–225. 10.1002/sia.740030506.

[ref46] Matanovic I.; Artyushkova K.; Atanassov P. Understanding PGM-Free Catalysts by Linking Density Functional Theory Calculations and Structural Analysis: Perspectives and Challenges. Curr. Opin. Electrochem. 2018, 9, 137–144. 10.1016/j.coelec.2018.03.009.

[ref47] Guo D.; Shibuya R.; Akiba C.; Saji S.; Kondo T.; Nakamura J. Active Sites of Nitrogen-Doped Carbon Materials for Oxygen Reduction Reaction Clarified Using Model Catalysts. Science 2016, 351 (6271), 361–365. 10.1126/science.aad0832. 26798009

[ref48] Behan J. A.; Iannaci A.; Domínguez C.; Stamatin S. N.; Hoque M. K.; Vasconcelos J. M.; Perova T. S.; Colavita P. E. Electrocatalysis of N-Doped Carbons in the Oxygen Reduction Reaction as a Function of PH: N-Sites and Scaffold Effects. Carbon 2019, 148, 224–230. 10.1016/j.carbon.2019.03.052.

[ref49] Chung H. T.; Cullen D. A.; Higgins D.; Sneed B. T.; Holby E. F.; More K. L.; Zelenay P. Direct Atomic-Level Insight into the Active Sites of a High-Performance PGM-Free ORR Catalyst. Science 2017, 357 (6350), 479–484. 10.1126/science.aan2255. 28774924

[ref50] Alves M. C. M. M.; Dodelet J. P.; Guay D.; Ladouceur M.; Tourillon G. Origin of the Electrocatalytic Properties for Oxygen Reduction of Some Heat-Treated Polyacrylonitrile and Phthalocyanine Cobalt Compounds Adsorbed on Carbon Black as Probed by Electrochemistry and x-Ray Absorption Spectroscopy. J. Phys. Chem. 1992, 96 (26), 10898–10905. 10.1021/j100205a054.

[ref51] Fei H.; Dong J.; Feng Y.; Allen C. S.; Wan C.; Volosskiy B.; Li M.; Zhao Z.; Wang Y.; Sun H.; An P.; Chen W.; Guo Z.; Lee C.; Chen D.; Shakir I.; Liu M.; Hu T.; Li Y.; Kirkland A. I.; Duan X.; Huang Y. General Synthesis and Definitive Structural Identification of MN4C4 Single-Atom Catalysts with Tunable Electrocatalytic Activities. Nat. Catal. 2018, 1 (1), 63–72. 10.1038/s41929-017-0008-y.

[ref52] Kocha S. S.; Shinozaki K.; Zack J. W.; Myers D. J.; Kariuki N. N.; Nowicki T.; Stamenkovic V.; Kang Y.; Li D.; Papageorgopoulos D. Best Practices and Testing Protocols for Benchmarking ORR Activities of Fuel Cell Electrocatalysts Using Rotating Disk Electrode. Electrocatalysis 2017, 8 (4), 366–374. 10.1007/s12678-017-0378-6.

[ref53] Bonakdarpour A.; Lefevre M.; Yang R.; Jaouen F.; Dahn T.; Dodelet J.-P.; Dahn J. R. Impact of Loading in RRDE Experiments on Fe-N-C Catalysts: Two- or Four-Electron Oxygen Reduction?. Electrochem. Solid-State Lett. 2008, 11 (6), B105 10.1149/1.2904768.

[ref54] Osmieri L.; Escudero-Cid R.; Armandi M.; Monteverde Videla A. H. A.; García Fierro J. L.; Ocón P.; Specchia S. Fe-N/C Catalysts for Oxygen Reduction Reaction Supported on Different Carbonaceous Materials. Performance in Acidic and Alkaline Direct Alcohol Fuel Cells. Appl. Catal., B 2017, 205, 637–653. 10.1016/j.apcatb.2017.01.003.

[ref55] Deng Y.; Dong Y.; Wang G.; Sun K.; Shi X.; Zheng L.; Li X.; Liao S. Well-Defined ZIF-Derived Fe-N Codoped Carbon Nanoframes as Efficient Oxygen Reduction Catalysts. ACS Appl. Mater. Interfaces 2017, 9 (11), 9699–9709. 10.1021/acsami.6b16851. 28244721

[ref56] Hossen M. M.; Artyushkova K.; Atanassov P.; Serov A. Synthesis and Characterization of High Performing Fe-N-C Catalyst for Oxygen Reduction Reaction (ORR) in Alkaline Exchange Membrane Fuel Cells. J. Power Sources 2018, 375, 214–221. 10.1016/j.jpowsour.2017.08.036.

[ref57] Specchia S.; Atanassov P.; Zagal J. H. Mapping Transition Metal-Nitrogen-Carbon Catalyst Performance on the Critical Descriptor Diagram. Curr. Opin. Electrochem. 2021, 27, 100687 10.1016/j.coelec.2021.100687.

[ref58] Osmieri L.; Monteverde Videla A. H. A.; Specchia S. The Use of Different Types of Reduced Graphene Oxide in the Preparation of Fe-N-C Electrocatalysts: Capacitive Behavior and Oxygen Reduction Reaction Activity in Alkaline Medium. J. Solid State Electrochem. 2016, 20 (12), 3507–3523. 10.1007/s10008-016-3332-2.

[ref59] Osmieri L.; Zafferoni C.; Wang L.; Monteverde Videla A. H. A.; Lavacchi A.; Specchia S. Polypyrrole-Derived Fe-Co-N-C Catalyst for the Oxygen Reduction Reaction: Performance in Alkaline Hydrogen and Ethanol Fuel Cells. ChemElectroChem 2018, 5 (14), 1954–1965. 10.1002/celc.201800420.

[ref60] Osmieri L.; Escudero-Cid R.; Monteverde Videla A. H. A.; Ocón P.; Specchia S. Application of a Non-Noble Fe-N-C Catalyst for Oxygen Reduction Reaction in an Alkaline Direct Ethanol Fuel Cell. Renewable Energy 2018, 115, 226–237. 10.1016/j.renene.2017.08.062.

