# Supplementary material for: Effect of the Thermal Treatment of Fe/N/C Catalysts for the Oxygen Reduction Reaction Synthesized by Pyrolysis of Covalent Organic Frameworks
Source: Ind Eng Chem Res. 2021 Nov 3;60(51):18759–69. doi: 10.1021/acs.iecr.1c02841 (PMC8719314; doi:10.1021/acs.iecr.1c02841)
Supplement: Supplementary file 1 — ie1c02841_si_001.pdf [file ie1c02841_si_001.pdf]

SUPPORTING INFORMATION

Effect of the thermal treatment of Fe/N/C catalysts for the oxygen reduction reaction synthesized by pyrolysis of covalent organic frameworks

Álvaro García,[1] Tarrick Haynes[1], María Retuerto,[1,*] Pilar Ferrer,[2] Laura Pascual,[4] Miguel A. Peña,[1] Mohamed Abdel Salam,[3] Mohamed Mokhtar,[3] Diego Gianolio,[2] Sergio Rojas[1,*]

[1]Grupo de Energía y Química Sostenibles, Instituto de Catálisis y Petroleoquímica, CSIC. Marie Curie 2, 28049, Madrid.

[2]Diamond Light Source, Harwell Science and Innovation Campus, Didcot, OX11 0DE, UK.

[3]Chemistry Department, Faculty of Science, King Abdulaziz University, P.O Box 80200, Jeddah, 21589, Saudi Arabia.

[4]Instituto de Catálisis y Petroleoquímica, CSIC. Marie Curie 2, 28049, Madrid.

*srojas@icp.csic.es, *m.retuerto@csic.es

1.1. XRD

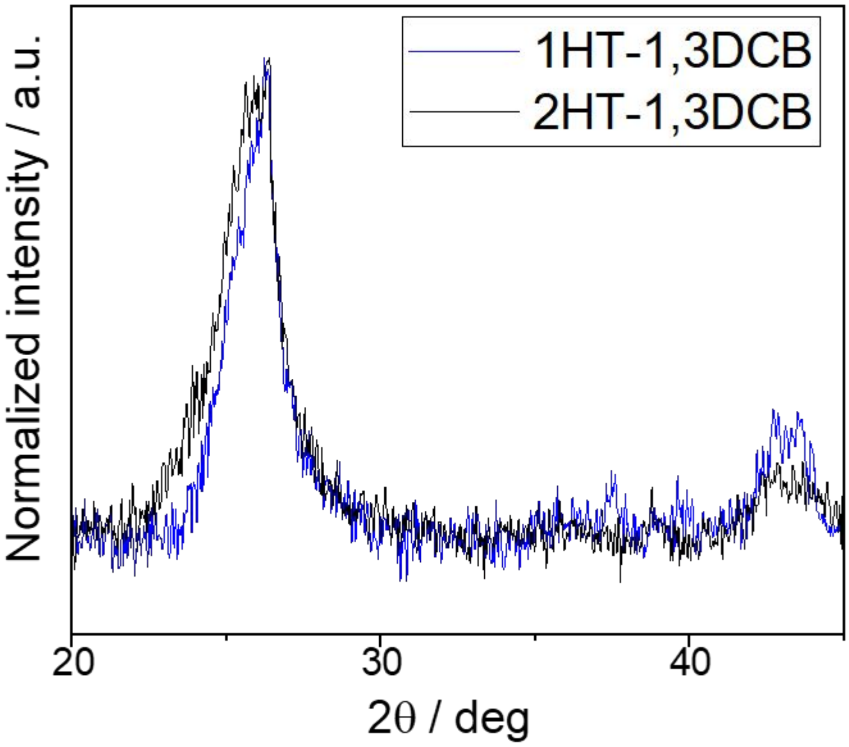

Figure S1. Magnification of the XRD showing the diffraction peaks for graphitic carbon at ca. 25.5 º and Fe3C at ca. 43.5º for samples 1HT-1,3DCB and 2HT-1,3DCB

1.2. X-Ray Photoemission Spectroscopy

C1s and Fe 2p core-level regions of 1HT-1,3DCB and 2HT-1,3DCB are shown in Figures S2 and S3 respectively. The C1s region are deconvoluted into 4 peaks, with a main component set at 284.6 eV due to graphitic carbon and the peak at 285.8 is included to account for the asymmetry of the main peak. Peaks at ca. 286.5 and 290.0 eV are ascribed to the presence of C–O and C=O species, respectively.

The Fe 2p core-level region display small peaks, especially in 2HT-1,3DCB, with the Fe $2p_{3/2}$ peaks at ca. 710 eV.

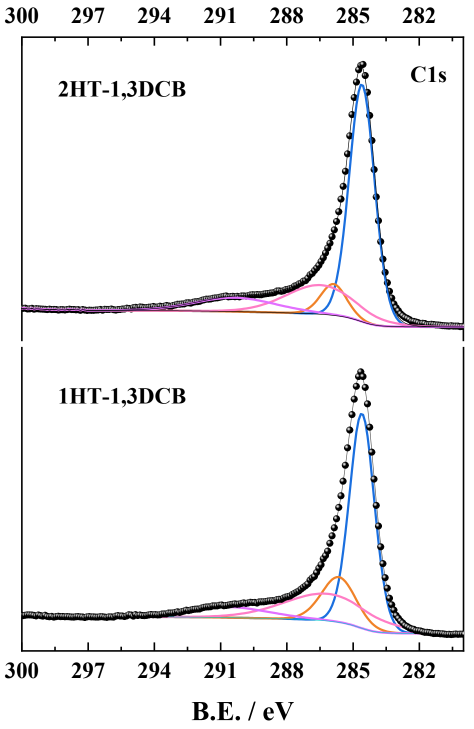

Figure S2. C 1s and core-level regions of the catalysts under study.

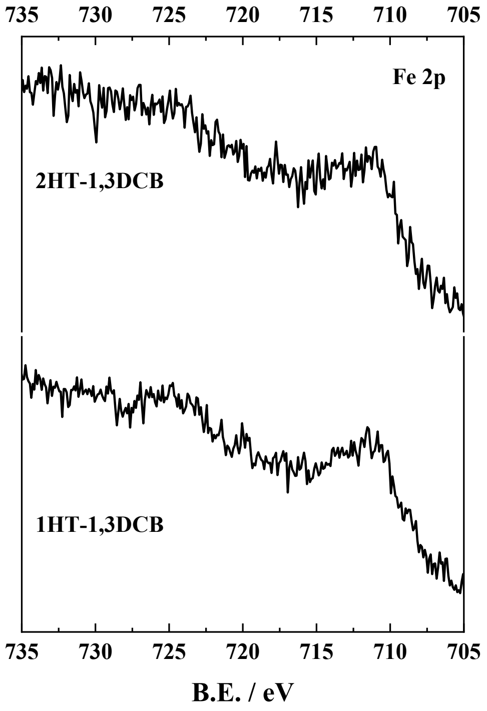

Figure S3. Fe 2p and core-level regions of the catalysts under study.

1.3. X-Ray Absorption Spectroscopy

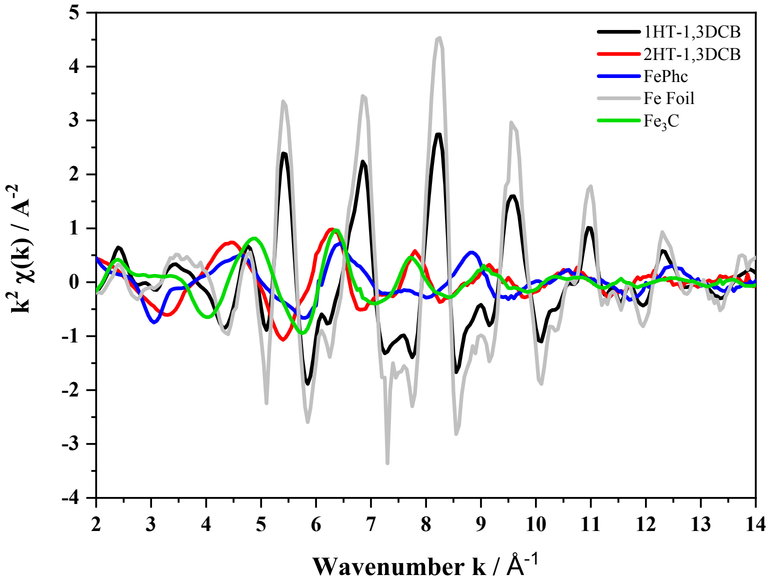

Figure S4. k-space EXAFS data of catalyst under study and standards.

1.4. Electrochemical performance: Accelerated Stress Test

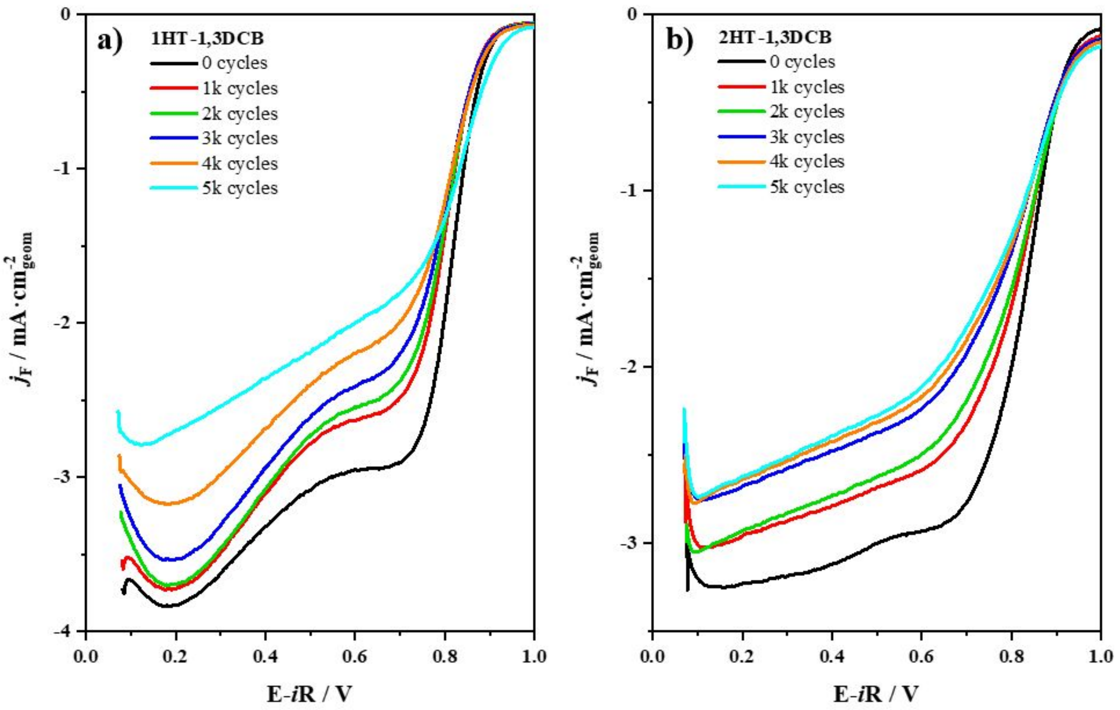

Figure S5. Load cycling accelerated stress test under oxygen saturated 0.1 M KOH electrolyte of a) 1HT-1,3DCB and b) 2HT-1,3DCB. Cycling sweeping recorded at 10 mVs^{-1} with rotating electrode at 1600 rpm.
